# Complement 3^+^-astrocytes are highly abundant in prion diseases, but their abolishment led to an accelerated disease course and early dysregulation of microglia

**DOI:** 10.1186/s40478-019-0735-1

**Published:** 2019-05-22

**Authors:** Kristin Hartmann, Diego Sepulveda-Falla, Indigo V. L. Rose, Charlotte Madore, Christiane Muth, Jakob Matschke, Oleg Butovsky, Shane Liddelow, Markus Glatzel, Susanne Krasemann

**Affiliations:** 1 Institute of Neuropathology, University Medical Center Hamburg-Eppendorf, Hamburg, Germany; 2 Neuroscience Institute; Neuroscience Institute, NYU Langone Medical Center, New York, USA; 3 Department of Neuroscience and Physiology, NYU Langone Medical Center, New York, USA; 4 Ann Romney Center for Neurologic Diseases, Department of Neurology, Brigham and Women’s Hospital, Harvard Medical School, Boston, MA USA; 5 Department of Pharmacology and Therapeutics, the University of Melbourne, Melbourne, Australia

**Keywords:** Prion diseases, A1-astrocytes, Microglia, Neurotoxicity

## Abstract

Astrogliosis and activation of microglia are hallmarks of prion diseases in humans and animals. Both were viewed to be rather independent events in disease pathophysiology, with proinflammatory microglia considered to be the potential neurotoxic species at late disease stages. Recent investigations have provided substantial evidence that a proinflammatory microglial cytokine cocktail containing TNF-α, IL-1α and C1qa reprograms a subset of astrocytes to change their expression profile and phenotype, thus becoming neurotoxic (designated as A1-astrocytes). Knockout or antibody blockage of the three cytokines abolish formation of A1-astrocytes, therefore, this pathway is of high therapeutic interest in neurodegenerative diseases. Since astrocyte polarization profiles have never been investigated in prion diseases, we performed several analyses and could show that C3^+^-PrP^Sc^-reactive-astrocytes, which may represent a subtype of A1-astrocytes, are highly abundant in prion disease mouse models and human prion diseases. To investigate their impact on prion disease pathophysiology and to evaluate their potential therapeutic targeting, we infected TNF-α, IL-1α, and C1qa Triple-KO mice (TKO-mice), which do not transit astrocytes into A1, with prions. Although formation of C3^+^-astrocytes was significantly reduced in prion infected Triple-KO-mice, this did not affect the amount of PrP^Sc^ deposition or titers of infectious prions. Detailed characterization of the astrocyte activation signature in thalamus tissue showed that astrocytes in prion diseases are highly activated, showing a mixed phenotype that is distinct from other neurodegenerative diseases and were therefore termed C3^+^-PrP^Sc^-reactive-astrocytes. Unexpectedly, Triple-KO led to a significant acceleration of prion disease course. While pan-astrocyte and -microglia marker upregulation was unchanged compared to WT-brains, microglial homeostatic markers were lost early in disease in TKO-mice, pointing towards important functions of different glia cell types in prion diseases.

**Electronic supplementary material:**

The online version of this article (10.1186/s40478-019-0735-1) contains supplementary material, which is available to authorized users.

## Introduction

Prion diseases are neurodegenerative disorders that are always fatal and affect humans and animals alike. Sporadic Creutzfeldt–Jakob disease (sCJD) is the most common human prion disorder. Prion diseases are characterized by the conformational conversion of the cellular prion protein PrP^C^ into the disease associated protein isoform PrP^Sc^ which is key to prion formation and disease progression [52]. Beside the accumulation of aggregated PrP^Sc^ in the brain, prion diseases are characterized by neuronal loss, spongiform lesions and widespread reactive gliosis. To date, the mechanisms of neurotoxicity leading to neuronal loss are only partially understood. While direct toxic signaling of misfolded PrP^Sc^ via cellular receptors on the neuronal membrane have been discussed [19, 55], non-neuron autonomous pathways have become of recent interest. The involvement of microglia in prion diseases was already noted decades ago [6, 24, 57]. Microglia are highly activated in prion disease mouse models and human prion diseases [34, 36]. Recent investigations have shown that microglia cells act beneficially at least in the early phases in in vitro*-* and mouse models of prion diseases [12, 49, 65]. However, it has been shown that suppression of microglia proliferation in the clinical disease phase significantly prolonged survival [22]. While microglia seem to be able to clear PrP^Sc^ at early disease stages, it was shown that microglia lose their PrP^Sc^ degrading function during disease progression [28]. Although microglia are the professional phagocytes of the brain, they get pro-inflammatory in the course of neurodegenerative diseases and may thereby contribute to neuronal loss in the late disease stages [1, 33].

Another hallmark of prion diseases is the widespread and severe reactive astrogliosis [45]. Since astrocyte activation was noted as a specific hallmark of prion diseases with significant up-regulation of glial fibrillary acidic protein (GFAP), the impact of its knockout on disease pathophysiology was tested several years ago [23, 59]. Interestingly, GFAP-knockout did not influence disease outcome [59].

Recently, it was shown that activation of microglia and astrocytes might not be as independent as assumed before: Liddelow at al. showed that a cytokine cocktail composed of TNF-α, IL-1α and C1qa that is released by activated microglia, could directly polarize a subset of astrocytes (designated A1-astrocytes) towards a neurotoxic phenotype [39]. This astrocyte subtype is characterized by increased expression of complement 3 (C3) as a typical marker [39, 40, 63]. While astrocytes in models of ischemic stroke may also express C3, however, this may rather correlate with a certain degrees of inflammation [64]. In contrast, astrocytes in scar formation seem to be devoid of C3 upregulation [18]. Of note, the genetic knockout or the targeting of TNF-α, IL-1α and C1qa with therapeutic antibodies was sufficient to abolish the formation of A1-astocytes after an appropriate stimulus in vitro and in vivo [39]. This, in turn, led to a better survival of neurons. Therefore, this detrimental pathway of microglia-to-astrocyte communication may be of high therapeutic potential in neurodegenerative diseases including prion diseases. Several proinflammatory cytokines are upregulated in the brain during the disease course in mouse prion disorders including TNF-α, IL-1α and C1qa [12, 29]. However, whether the activation of microglia might lead to the formation of A1-astrocytes that affect neuronal survival and disease progression in prion disorders was never assessed before.

Therefore, we wanted to investigate if A1-like-astrocytes are abundant in prion diseases, how they might affect prion disease pathophysiology, and if their abolishment might be a therapeutic option for treatment. Using specific antibodies, we could show that C3^+^-astrocytes are highly abundant in a prion disease mouse model and in human sCJD. We then investigated the impact of these astrocytes on prion disease pathophysiology by prion infecting mice with a knockout of the three cytokines TNF-α, IL-1α and C1qa, which are unable to develop A1-astrocytes upon stimulation. We assessed PrP^Sc^ loads, titer of infectious prions as well as astrocyte and microglia activation markers at different time points during the course of disease. Although the deposition of misfolded PrP^Sc^ was unchanged, we found that knockout of TNF-α, IL-1α and C1qa with the abolishment of C3^+^-astrocyte formation let to a significant acceleration of the prion disease course. This was paralleled by early dysregulation of homeostatic microglia profile. Our data rather exclude the abolishment of C3^+^-astrocytes as a therapeutic strategy in prion diseases.

## Material and methods

### Ethics statement

All animal experiments were approved by the Ethical Committee of the Freie und Hansestadt Hamburg, Amt für Gesundheit und Verbraucherschutz (Permit number: V 1300/591–00.33) and in strict accordance with the principles of laboratory animal care (NIH publication No. 86–23, revised 1985) and the recommendations in the Guide for the Care and Use of Laboratory Animals of the German Animal Welfare Act on protection of animals. All applicable international, national, and/or institutional guidelines for the care and use of animals were followed. All inoculations were performed under Ketamine and xylazine hydrochloride anaesthesia, and all efforts were made to minimize suffering. Mice received a single intraoperative injection of Rimadyl (Carprofen 6 mg/kg) for post-operative pain prophylaxis.

Ethical approval for the use of anonymized human post mortem tissues was obtained from the Ethical Committee at the University Medical Center Hamburg-Eppendorf and is in accordance with ethical regulations at study centers and with the 1964 Helsinki declaration and its later amendments or comparable ethical standards.

### Chemicals

Chemicals were purchased from Sigma-Aldrich (St.Louis, USA), if not otherwise indicated.

### Animals

C57/Bl6-mice were purchased from Charles River/Germany. Triple-KO-mice (knockout of TNF-α, IL-1α and C1qa on a C57/Bl6 background) where provided by Shane Liddelow [39]. Eight weeks old male and female mice of both groups were intra-cerebrally inoculated with brain homogenate from terminally RML 5.0-prion infected mice (3 × 10^5^ logLD_50_ (high dose)). Control animals received mock homogenate (brain homogenate from uninfected CD-1 mice). Mice were taken at preclinical days 80 and 110 post prion injection. To determine the incubation time to clinical prion disease, remaining mice were allowed to progress to terminal prion disease, where brain tissue was collected and processed either for immunohistochemistry or stored at − 80 °C for biochemical analyses. Terminal prion disease stage was determined blinded to the mouse genotype by an independent researcher. Control mice were taken at corresponding time points for analysis.

### Human CJD cases

We analyzed post-mortem brain tissue samples that were obtained through Reference Center activities of the German National Reference Center for Surveillance of Transmissible Spongiform Encephalopathies and the National Reference Center for Prion Diseases of the German Society of Neuropathology (see Table 1 for overview). Control tissues were obtained post-mortem from the University Medical Center Hamburg-Eppendorf and were age and gender matched for the investigated CJD cases (Table 2). All cases underwent standardized neuropathological assessment, including macroscopic and microscopic examination. Controls did not show any sign of neurologic or neurodegenerative diseases.

**Table 1:** Summary of clinical parameters of Creutzfeldt-Jakob patients enrolled with post mortem brain tissue samples in this study

Age	Sex	Diagnosis	Subtype	Disease duration [months]	14–3-3	RT-Quic
F	69	CJD	VV1	9	+	+
M	72	CJD	MM/MV1	n.a.	n.a.	n.a.
F	78	CJD	MM/MV1	n.a.	n.a.	n.a.
F	59	CJD	MV2K	45	n.a.	n.a.
M	78	CJD	MV2K + C	10	–	n.a.

**Table 2:** Summary of age and gender matched human post mortem control brain samples

Age	Sex	Diagnosis	Cause of death
F	69	Control	Bronchogenic adenocarcinoma
M	72	Control	Aortic dissection
F	78	Control	Acute myocardial infarction
F	59	Control	Cryptogenic liver cirrhosis
M	79	Control	Ventricular fibrillation

### Determination of prion titer by bioassay

To determine the content of infectious prions in a given tissue, 1% tissue homogenate (0.3 μg) was inoculated intra-cerebrally into groups of 4 PrP^C^-overexpressing *tg*a*20* transgenic mice [20]. Animals were observed daily and sacrificed when clinical signs of prion disease (reduced motor activity, weight loss, hunched posture, hind limb paresis, and ataxia) were evident. Prion titers were calculated according to the following equation (y = 11.45–0.088x), where x is the incubation time to terminal disease in days and y is LD_50_ [20].

### Western blot analysis

For Western blot analysis, brains were homogenised (FastPrep FP120, Qbiogene, Illkirch, France) at 10% (weight/volume, w/v) in RIPA buffer (150 mM NaCl, 1% NP-40, 0.5% DOC, 0.1% SDS, 50 mM Tris-HCl pH 8.0) and a subset of samples from comparable time points were digested with proteinase K (PK) (20 μg/ml) (Roche, Mannheim, Germany) for 1 h at 37 °C. Digestion was stopped by addition of 10× sample buffer and boiling for 10 min. Samples were analyzed by SDS-page (AnykD, Biorad, Hercules, USA), transferred to nitrocellulose membranes (0.2 μm pore size, BioRad) at 400 mA for 1 h, blocked for 1 h at room temperature in 5% milk powder in TBST buffer and incubated overnight at 4 °C with anti-PrP antibody Pom1 [50], Iba1 (Wako), Actin (Millipore), and GLP-1R (Santa Cruz) [63]. After washing and incubation for 1 h at room temperature with an HRP-conjugated anti-mouse or anti-rabbit secondary antibody (1:10.000 in blocking buffer), signals were detected with ECL femto reagent (Thermo Scientific) and visualized and quantified with a BioRad ChemiDoc imaging station and Biorad VersaDoc.

### Immunohistochemistry

Mouse brain tissues were fixed in 4% buffered formalin and prion infectivity was inactivated by immersion in 98% formic acid for 1 hour. Human brain tissues were fixed in 4% buffered formalin. To inactivate prion infectivity, tissues were incubated in 98% formic acid for 1.5 h. Tissues from control mice and healthy human controls were treated with formic acid, too, to enable identical staining conditions. Mouse tissues were rinsed thoroughly, post fixed in 4% buffered formalin overnight and processed for paraffin embedding. Alternatively, tissues were soaked in 20% sucrose/PBS overnight, embedded in Tissue Tek, frozen into blocks and stored at − 80 °C. Sections (2 μm for paraffin, 8 μm for frozen tissue) were subjected to HE staining and glial fibrillary acidic protein (GFAP; Dako), ALDH1L1 (Abcam), ionized calcium binding adaptor molecule 1 (Iba1; Wako), YKL-40 (Thermo Fisher Scientific), microglial homeostatic markers TMEM119 (Synaptic Systems), and P2ry12 [11] immunohistochemistry according to standard protocols using a Ventana Benchmark XT (Ventana, Tuscon, Arizona, USA). Antigen retrieval was performed on deparaffinised sections by boiling for 30 to 60 min in 10 mM citrate buffer, pH 6.0. Sections were incubated with primary antibody for 1 h, anti-rabbit or anti-mouse Histofine Simple Stain MAX PO Universal immunoperoxidase polymer (Nichirei Biosciences, Wedel, Germany) were used as secondary antibodies. Detection of secondary antibodies and counter staining was performed with an ultraview universal DAB detection kit from Ventana (Ventana, Tuscon, Arizona, USA). Data acquisition was performed using a Leica DMD108 digital microscope. Positive signal area and particle size (cellular bodies or processes) were quantified in three to four different sections for GFAP, Iba1, P2ry12 and TMEM119 using the Analyze function in the ImageJ 1.52e software [56]. Total area for each section was 273,000 μm^2^.

For the profiling of spongiform lesions from each experimental group at least 3 mice were analyzed by a scientist that was blinded to the animal identity, at 4 different anatomical regions: cortex, hippocampus, thalamus, and cerebellum. Spongiosis was scored in HE-sections on a scale of 0–4 (not at all, mild, moderate, severe, status spongiosus). Since the degree of spongiosis was still low in preclinical animals, for assessment of days post infection 80 and 110, the sum of the scores of all four brain regions per animal was plotted. However, at clinical disease, we displayed the lesion pattern score of every brain region separately.

Human paraffin embedded tissues were cut at 2 μm sections and stained similar as the mouse tissues using primary antibodies GFAP (Dako), YKL-40 (Thermo Fisher Scientific), Complement 3 (Abcam), and GBP2 (LSBio). For PrP^Sc^-detection in human brains, mounted paraffin tissue sections (3 μm) were incubated at least overnight at 60 °C. Sections were deparaffinized and boiled for 30 min in 2 mM hydrochloric acid. After cooling down, sections were pretreated with 98% formic acid for 5 min. Further processing was performed on an automated staining machine (BenchMarkTX, VENTANA, Roche Diagnostics, Mannheim, Germany) without further pretreatment. PrP^Sc^ was detected with anti-prion antibody 3F4 (Merck), followed by the secondary antibody biotinylated anti-mouse IgG. Color development and counterstaining was according to standard protocols.

### Immunofluorescence analysis

Human formic acid inactivated and paraffin embedded tissues were cut into 2 μm sections. These were dewaxed and antigen retrieval was performed for 30 min at 96 °C in 10 mM citrate buffer pH 6.0. Mouse tissues were likewise inactivated in 98% formic acid, washed, incubated in 20% sucrose overnight, embedded in tissue tek, frozen, and cut at 8 μm thickness on a cryostat. Sections were washed, permeabilized with 0.2% TritonX 100 (Roche) in TBS and blocked in blocking buffer (Protein-Free T20 (TBS) Blocking buffer #37071 Thermo Fischer) for 1 h. A1 astrocyte marker anti-Gbp2 antibody (LSBio) and anti-GFAP (Dako) or anti-Iba1 (Synaptic Systems) were applied on the human sections, A1-astrocyte marker C3d (R&D Systems) [63], A1-astrocyte marker C3 (HycultBiotech) [40], and anti-GFAP (Chemicon) on the mouse sections overnight at 4 °C with gentle agitation. Afterward, sections were intensively washed and followed by incubation with Alexa647-conjugated secondary anti-rabbit antibody and Alexa488-conjugated secondary anti-mouse antibody or A488-conjugated secondary anti-guinea pig antibody for 1.5 h at room temperature. After repeated washing, sections were mounted with Fluoromount-G (SouthernBiotech, Birmingham, USA). Data acquisition was performed using a Leica Sp5 confocal microscope and Leica application suite software (LAS-AF-lite). Positive signal area was quantified in three different sections for GFAP and C3 using the Analyze function in the ImageJ 1.52e software [56]. Total area for each section was 62.500μm^2^.

### RNA isolation and quantitative real-time PCR

Total RNA was extracted from mouse thalamus brain tissue using the miRCURY™ RNA Isolation Kit (Exiqon; Cell and Plant #300110) according to the manufacturer with one exception: To inactivate prion infectivity, tissues were homogenized in lysis buffer and incubated in it for 2 h at room temperature before further processing of the RNA.

Total RNA (30 ng) with specific mRNA probes (Applied Biosystems) were used for conventional quantitative reverse transcription polymerase chain reaction (qRT-PCR), after reverse transcription reaction according to the manufacturer (high-capacity cDNA Reverse Transcription Kit; Applied Biosystems). Amplifications were performed using Vii7 (Applied Biosystems) with commercially available FAM-labeled Taqman probes (Applied Biosystems/Thermo Fisher Scientific) and mRNAs levels were normalized relative to GAPDH. All qRT-PCRs were performed in duplicate, and the data are presented as relative expression compared to *Gapdh* as mean ± s.e.m.

### mRNA analysis using microfluidics qPCR

Total RNA was extracted from whole brain, from wildtype (WT) or Il1a−/−Tnf−/−C1qa−/− triple knock-out (TKO) animals following prion infection or saline injection. Total RNA was extracted using the qScript™ cDNA SuperMix kit (QuantaBio). We designed primers using NCBI primer Basic Local Alignment Search Tool (BLAST) software, and as described previously all primers had 90 to 105% efficiency, primer pairs to amplify products that spanned exon–exon junctions to avoid amplification of genomic DNA, and specificity of primer pairs was examined using agarose gel electrophoresis [39]. Samples were prepared as previously described [39] and involved preamplification for genes of interest, removal of excess primers and dilution of sample. Five microliters of sample mix containing preamplified cDNA and amplification Master mix (20 mM MgCl2, 10 mM dNTPs, FastStart Taq polymerase, DNA-binding dye loading reagent, 50× ROX, 20× Evagreen) was loaded into each sample inlet of a 96.96 Dynamic Array chip (Fluidigm Corporation), and 5 μL from an assay mix containing DNA-assay loading reagent, as well as forward and reverse primers (10 pmol·μL − 1) was loaded into each detector inlet. Dynamic Array Chips were mixed and loaded using a Nano-FlexTM 4-IFC Controller (Fluidigm) before processing the chip in a BioMark HD Real-Time PCR System (Fluidigm) using the standard fast program. Data were collected using BioMark Data Collection Software 2.1.1 build 20,090,519.0926 (Fluidigm) as the cycle of quantification, where the fluorescence signal of amplified DNA intersected with background noise. Fluidigm data were corrected for differences in input RNA using the mean of the reference gene Rplp0. Data preprocessing and analysis was completed using Fluidigm Melting Curve Analysis Software 1.1.0 build 20,100,514.1234 (Fluidigm) and Real-time PCR Analysis Software 2.1.1 build 20,090,521.1135 (Fluidigm) to determine valid PCR reactions. Invalid reactions were removed from later analysis.

### mRNA analyses using nanoStringTM nCounter®

Total RNA was extracted as above, and purity and concentration measured using a NanodropTM OneC microvolume UV-Vis spectrophotometer (ThermoFisher). One hundred nanograms of total RNA was used to run an nCounter® Inflammation Panel (Mouse v2) to detect 770 mRNA targets, with additional custom targets included (Aldh1l1, Gfap, Aspg, Ggta1, H2-D1, Hsbp1, Iigp1, Stat3). Chips were run by the Genome Technology Center at NYU Langone School of Medicine on an nCounter® MAX Analysis System (nanoStringTM Technologies). Gene expression was normalized using the included 30 predefined reference genes using the nSolver Anlaysis Software (v4.0, nanoString Technologies). Heatmaps were generated using the ClustVis online tool [46].

### Statistical analyses

For statistical comparison of data the Graphpad Prizm v7.05 program was used applying one-way ANOVA with Tukey’s multiple comparison for grouped analyses. For comparison of two cohorts, student’s t-Test was applied. For Kaplan-Meier curve calculation the Log-rank (Mantel-Cox) test was applied and for immunohistochemical-based quantifications a semi-quantitative measurement was applied, with sample identity blinded to the investigator. A minimal significance value was determined when *p* < 0.05. Individual analyses and significance are described in each figure legend. Levels for statistical significance were set at *p*-values < 0.05 (*), < 0.01 (**) and < 0.005 (***).

## Results

### A1 astrocytes are highly abundant in mouse and human prion diseases

**Fig. 1:**
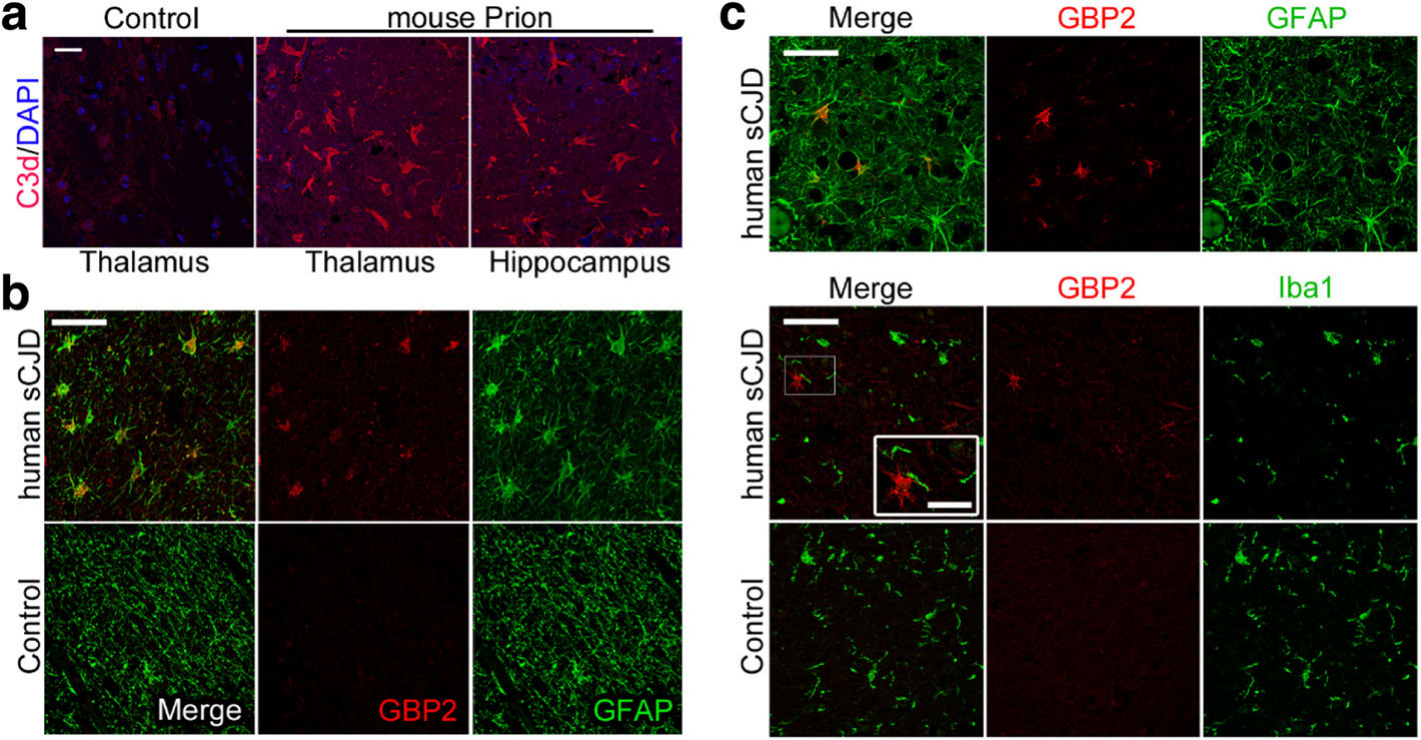
A1-astrocytes are highly abundant in human prion diseases and a prion mouse model. **a** Representative immunofluorescence staining with the A1-astrocyte marker C3d showed high abundance of A1 astrocytes in different brain regions in terminally prion diseased mice (*n* = 3 individual animals per group), (age matched control mice; n = 3); red = C3d; blue = DAPI; scale bar: 25 μm. **b** Representative frontal cortex sections of control or sCJD individuals showed that GBP2-positive A1-astrocytes can be only detected in human prion disease brains. GBP2-signal co-localize with GFAP-positive astrocytes; (CJD cases; *n* = 5), (age and gender matched control; n = 5); red = GBP2; green = GFAP; scale bar: 50 μm. **c** Representative fluorescence staining of human frontal cortex brain sections show that Iba1-positive microglia do not co-localize with GBP2, but keep close contact with GBP2^+^ astrocytes (see high magnification insert); GFAP (green) or Iba1 (green) and GBP2 (red); scale bar: 50 μm, close up: 20 μm

Reactive astrogliosis is a hallmark of human neurodegenerative diseases, however, it is particularly highly abundant in prion disease mouse models and human prion diseases [41, 45]. Human sCJD prion diseased patients show a highly positive staining pattern for the pan-astrocyte markers GFAP and YKL-40 in brain tissue (Additional file 1: Figure S1a). Brain tissues of terminally sick RML5.0-prion infected mice were likewise highly positive for pan-astrocyte markers GFAP and YKL-40 (Additional file 1: Figure S1a). Positive staining in the mouse brain is highly prevalent especially in areas with high amount of PrP^Sc^ deposition as in thalamus. To evaluate, if A1-astrocytes are abundant in terminally sick mice, we stained brain sections with the A1-marker complement 3 (C3) (Fig. 1a) [39, 63]. Immunohistochemistry for C3d showed positive cells in mice infected with prions, but not in control animals, suggesting that prion infection is able to activate astrocytes down this neurotoxic phenotype (Fig. 1a). To assess, if formation of A1-astrocytes also plays a role in human prion diseases, we stained human brain tissue sections for the putative A1-astrocyte markers C3 (Additional file 1: Figure S1b) and guanine-binding protein 2 (GBP2) (Fig. 1b, c, Additional file 1: Figure S1b) [39]. Human sCJD prion disease cases showed C3-positive astrocytes in comparison to control cases (Additional file 1: Figure S1b). However, non-astrocytic background staining was high with C3-specific antibody in the human samples (Additional file 1: Figure S1b). In contrast, GBP2 was highly positive and co-localized with GFAP^+^-astrocytes in human sCJD (Fig. 1b). Of note, GBP2-positive astrocytes were completely absent in human age- and gender-matched healthy control individuals (see Additional file 1: Figure S1c, d). Therefore, GBP2 seems highly suitable to identify specifically polarized astrocytes in human brain tissue. Interestingly, abundance of GBP2-positive-astrocytes was completely independent of the amount of pathological prion protein deposition which was highly variable in the investigated cases (for individual PrP^Sc^ deposition pattern see Additional file 1: Figure S2). To confirm specific co-localization of GBP2 with astrocytes, we performed double-immunofluorescence staining of GBP2 and GFAP or microglial marker Iba1, respectively (Fig. 1c). While GBP2 immunoreactivity almost exclusively co-localized with GFAP^+^-astrocytes, Iba1^+^-microglia did not co-localize with GBP2 at all (Fig. 1b, c). Interestingly, microglia processes could be detected in close contact with GBP2^+^/GFAP^+^-A1-astrocytes (close-up in Fig. 1c). We detected a small number of GBP2-positive oligodendrocytes or oligodendrocyte precursor cells (data not shown). However, those were also only detectable in prion disease sCJD cases, but not in control individuals.

### Triple cytokine knockout does not influence PrP^Sc^-formation but leads to acceleration of prion disease course

**Fig. 2:**
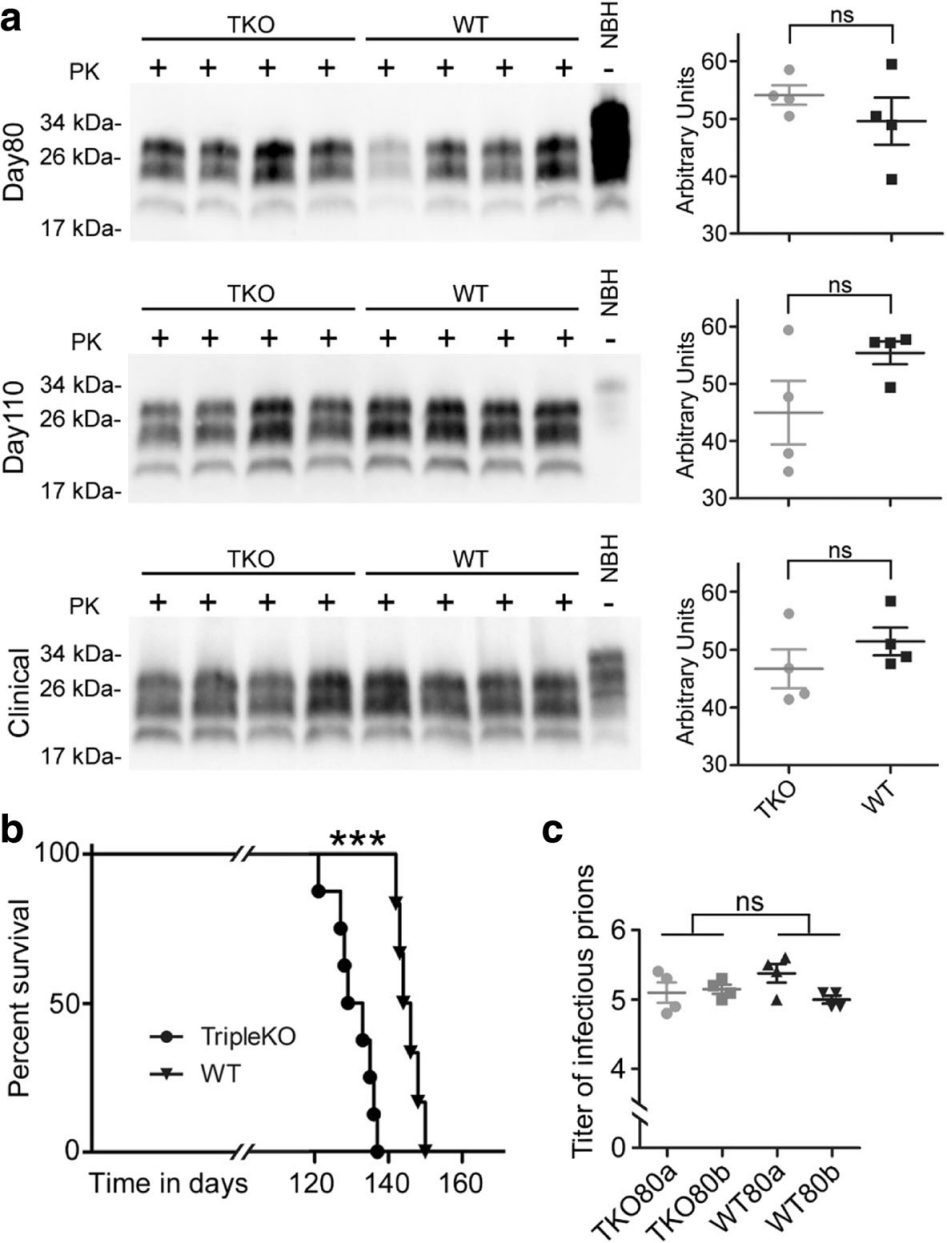
Triple-KO leads to significant acceleration of prion disease course **a** Western blot analysis of PrP^Sc^ after proteinase K digestion of brain tissue at 80 and 110 days p.i. and at clinical prion disease. Quantification of signal intensity showed that WT and Triple-KO mice do not show significant differences at all three investigated time points (*n* = 4 independent animals per group and time point) day80 *p* = 0.347; day 110 *p* = 0.126; clinical prion disease *p* = 0.297. **b** Kaplan-Meier survival curve of RML-prion infected WT- (*n* = 8 individual animals) and TKO-mice (*n* = 10 individual animals), Mantel-Cox log rank *** *p* = 0.0003. **c** Titers of prion infectivity as measured by bioassay in *tga*20-mice are similar in brains of WT- or TKO-mice 80 days post prion infection (*p* = 0.1451). Brain homogenates of two individual infected mice per group were injected into 4 individual tga20-mice, each

**Fig. 3:**
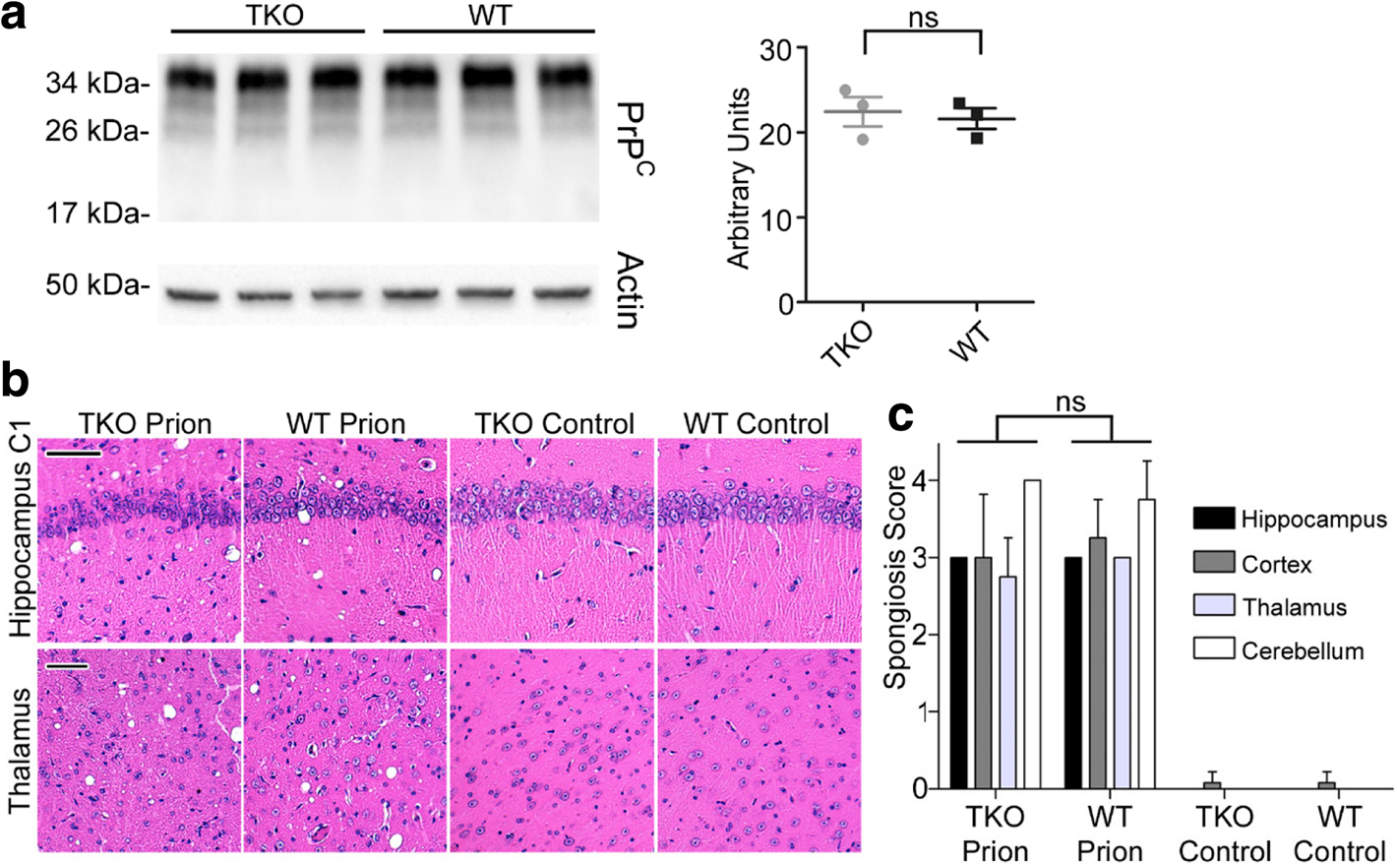
Prion pathology in TKO- is similar to WT-mice at clinical time points **a** Determination of PrP^C^ expression by Western blot and subsequent quantification of PrP^C^ normalized to β-actin in age matched non-infected mice brain homogenates showed similar expression levels at the age of 200 days (corresponding to the clinical time point). **b** Representative H&E staining of hippocampus and thalamus of clinical prion diseased mice and age matched non-infected controls; scale bar: 50 μm. **c** Semi-quantitative determination of spongiosis levels showed no differences between clinical TKO versus WT mice (n = 4 individual mice each). In contrast, uninfected TKO or WT mice do not display spongiosis (*n* = 3 individual mice each)

We show that C3^+^-astrocytes are highly abundant in prion diseases. Since A1-astrocytes are considered to exert neurotoxic functions and might thereby influence disease progression, we wanted to investigate their impact in the prion disease mouse model. Therefore, we intra-cerebrally infected Triple-KO mice (TKO) lacking expression of TNF-α, IL-1α and C1qa (which fail to develop A1 reactive astrocytes following inflammatory insult [39]) with RML-prions and determined pathophysiology of prion disease progression in comparison to infected WT-mice (WT). We took mice at preclinical days 80 and 110 post infection (p.i.) and let the third group of mice progress to terminal prion disease. Amounts of pathological prion protein PrP^Sc^ were determined by Western-blot analysis after PK-digestion of brain lysates, but did not differ between experimental groups at 80, 110 p.i. and at clinical time points (Fig. 2a). Unexpectedly, TKO-mice displayed a significantly accelerated prion disease time course (*p* = 0.0003) (Fig. 2b; Additional file 1: Table S1). While mean survival of WT-mice was 146 +/− 3 days, TKO-mice had to be sacrificed due to terminal disease at 131 +/− 5 days. Since, it was shown that the amounts of deposition of misfolded PrP^Sc^ and the titer of infectious prions in tissue are not necessarily comparable [7, 35], we determined, if titers of infectious prions were responsible for the differences in incubation time. For this, we performed bioassays via injection of brain tissues of TKO versus WT-mice at day 80 p.i. into highly susceptible *tga*20-mice (Fig. 2c). However, titers of infectious prions were comparable in both groups (*p* = 0.1451). To rule out the possibility that shortening of incubation time in TKO-mice is due to differences in PrP^C^ expression, a prerequisite for PrP^Sc^ formation and pathology [9, 10], we performed Western Blot analyses of PrP^C^ at different corresponding time points from non-infected animals. However, PrP^C^ protein levels were similar in WT- and TKO-mice (Fig. 3a).

To assess neuropathological changes in the experimental groups, we stained brain sections of terminally sick mice with H&E and determined the degree of spongiosis by semi-quantitative measurement (Fig. 3b). Although both cohorts, WT and TKO, showed increased levels of spongiosis in different regions of the brain compared to non-infected mice of each genotype, they were unchanged in TKO-mice. When we assessed astrocytes distribution and morphology at clinical time points by staining with the pan-astrocyte marker GFAP, it was highly upregulated upon prion infection. To our surprise, we could not detect changes in immuno-histochemical staining between prion-infected TKO- or WT-mice (Additional file 1: Figure S3, S4). Since using GFAP as a sole astrocyte marker might not be sufficient, we also investigated immunoreactivity of two other astrocyte proteins: YKL-40 and ALDH1L1 (Additional file 1: Figure S3). The latter were both increased in their intensity upon clinical prion disease. However, as with GFAP, we could not detect differences in the astrocyte activation profiles of infected WT- versus TKO-mice (Additional file 1: Figure S3). Interestingly, while GFAP was more homogenously upregulated in brains of infected animals, YKL-40 and ALDH1L1 showed more prominent increase in thalamus, the region with highest PrP^Sc^ deposition. The pan-microglia/monocyte marker Iba1 was likewise increased in terminal prion infected animals but did not differ between TKO- or WT-mice (Additional file 1: Figure S5). In contrast, the microglia homeostasis marker TMEM119 was downregulated upon prion disease in both cohorts (Additional file 1: Figure S5). Next, we determined amount of microglial Iba1 expression via Western blot analysis at day 80, day 110 post infection and at clinical prion disease. Although Iba1 was significantly upregulated in prion diseased animals, again, we could not detect changes between TKO- and WT-mice upon prion infection (Additional file 1: Figure S6).

### Astrocyte expression signature is specifically changed in prion disease

**Fig. 4:**
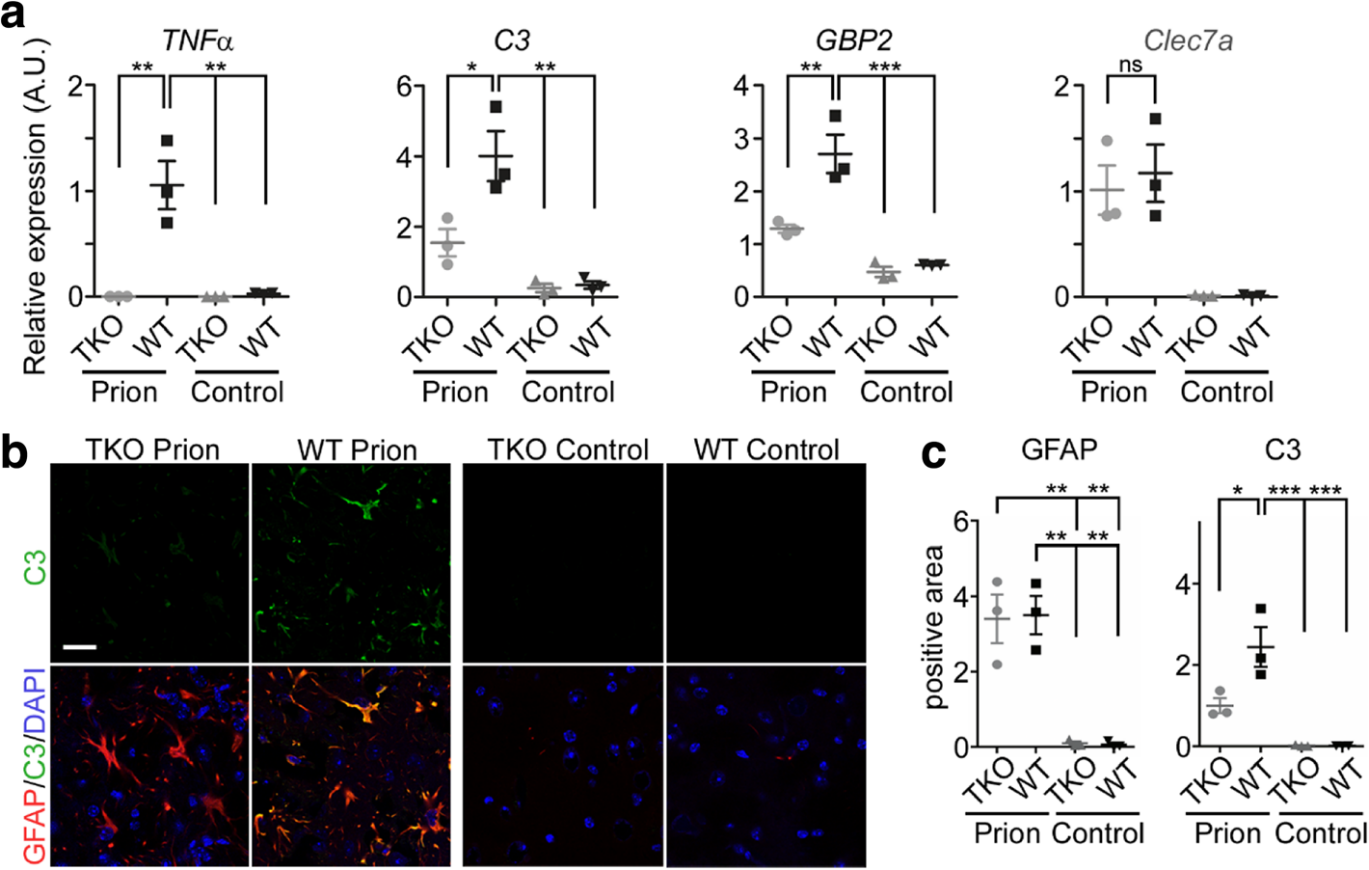
A1-astrocyte markers are significantly altered in TKO mice upon prion infection **a** qPCR expression analysis of microglia and astrocyte disease markers in thalamus tissue from terminally sick mice and age matched control confirmed the knockout of TNFα in TKO mice, but significant upregulation in diseased WT-mice (*p* = 0.0004). A1-astrocyte markers are significantly less upregulated in diseased TKO-mice (C3 *p* = 0.0006; GBP2 *p* = 0.0001). In contrast, microglia disease marker Clec7a was upregulated in both infected groups (*p* = 0.0023) n = 3 independent mice/group. **b** Representative staining of GFAP (red), C3 (green) and DAPI (blue) in Thalamus tissue at clinical prion disease. **c** Quantification of positive staining area (μm^2^ × 1000) showed significant upregulation of GFAP in both, prion infected WT- and TKO-mice (p = 0.0003), while C3 is significantly upregulated in WT-mice only (*p* = 0.0005); n = 3 independent from Fig. 4a animals/group

We next assessed expression levels of A1-astrocyte markers as well as microglia disease markers via qPCR (Fig. 4a). We first assessed expression of *TNF-α* as an internal control. *TNF-α* was significantly upregulated upon terminal prion disease in brains of WT animals, but as expected, not abundant in the TKO-mice. In contrast to the pan-astrocyte maker GFAP, which was highly upregulated in both infected groups (Additional file 1: Figure S3, S4), *C3* and *GBP2* were both significantly upregulated in terminally sick WT-animals, but not in the TKO-mice, as would be expected when devoid of formation of A1-astrocytes (Fig. 4a). However, the microglia disease marker *Clec7a* [33] was significantly upregulated in both groups of infected animals (Fig. 4a). To confirm the differences in C3 protein levels, we performed double staining of C3 and GFAP in brains of another set of terminally sick mice (Fig. 4b). While GFAP immunoreactivity was unchanged between prion diseased TKO- and WT-mice, C3 was significantly less upregulated in terminally sick TKO-mice (Fig. 4c).

**Fig. 5:**
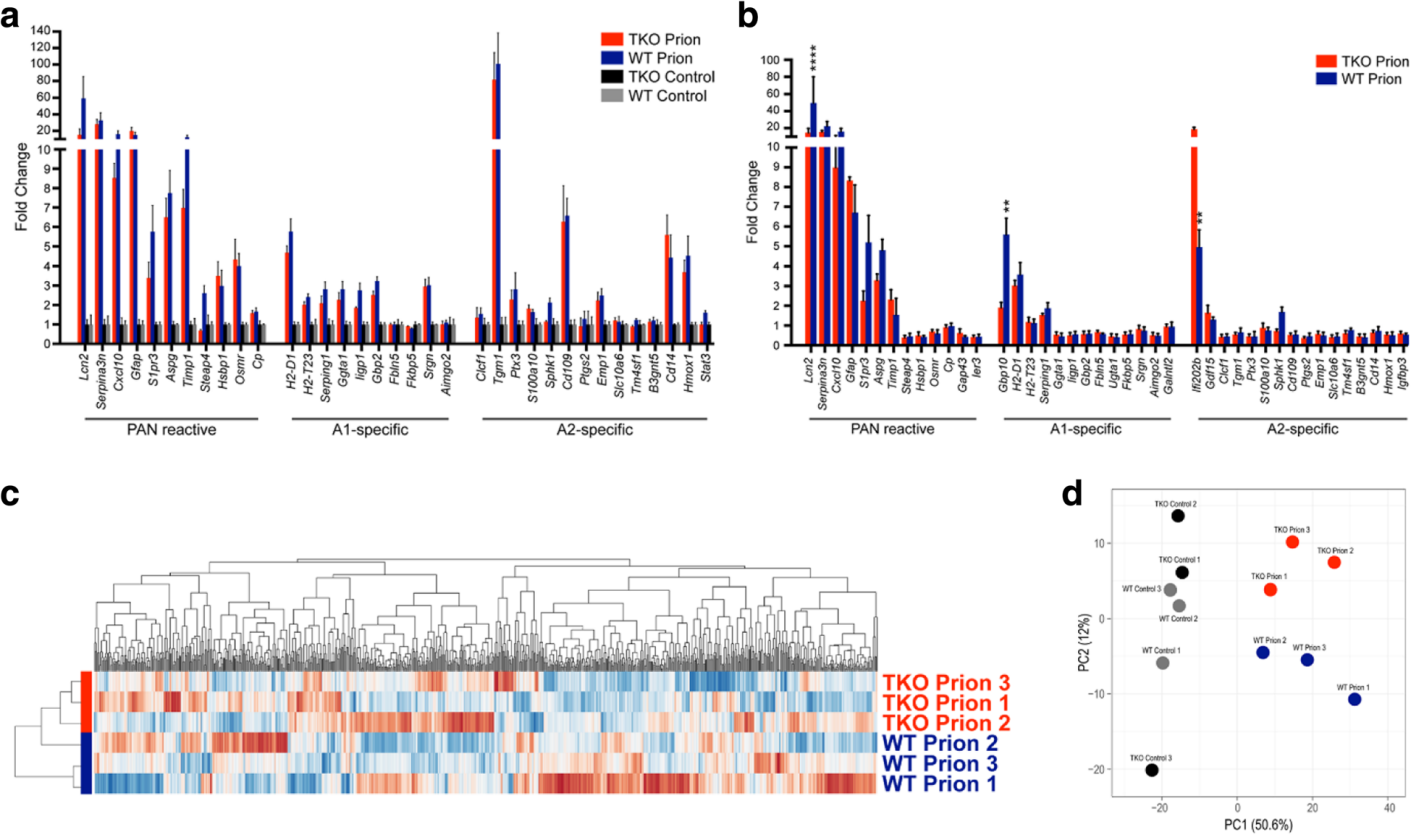
Analyses of immune cell and astrocyte marker expression. **a** Fold change induction of reactive astrocyte specific transcripts split to Pan-, A1-, and A2-specific cassettes for all experimental groups including age matched control groups. Data are mean +/−SEM using Nanostring nCounter data. **b** Fold change induction of reactive astrocyte specific transcripts split to Pan-, A1-, and A2-specific cassettes showing significant differences between infected WT- and TKO-mice. Data are mean +/−SEM using microfluidic qPCR. **c** Heatmap of transcripts of Nanostring encounter expression analysis from terminally sick prion infected WT- and TKO-mice. Although subtle, there are clustering differences between infected WT- versus TKO-mice. **d** PCA plot of Nanostring expression data. Unit variance scaling is applied to rows; singular value decomposition with imputation is used to calculate principal components. X and Y axis show principal component 1 and principal component 2 that explain 50.6 and 12% of the total variance, respectively. All control animals regardless of genotype cluster closely together, while prion infected animals are clustered according to genotype; n = 3 individual animals per group for all analyses

To investigate the astrocyte activation profile at clinical prion disease in more detail, we performed microfluidic qPCR [39] and Nanostring expression analysis using bulk RNA from thalamus tissue of infected terminally sick animals and age matched controls of both genotypes. Analysis of expression of specific Pan-, A1- and A2-astrocyte marker proteins revealed that in terminal prion disease, a mixed astrocyte activation phenotype is generated (Additional file 1: Figure S7a, b). Astrocyte profiles are characterized by significant and high upregulation of pan-markers and a mixed upregulation of A1 and A2-markers (Fig. 5a, b; Additional file 1: Figure S7a, b). Therefore, reactive astrocytes in prion diseases might be termed C3^+^-PrP^Sc^-specific to distinguish them from classical A1-astrocytes in other neurodegenerative diseases. Although subtle, astrocyte activation signatures differ between terminally sick WT- and TKO-mice, which cluster separately (Fig. 5b; Additional file 1: Figure S7b). This is even more obvious when including other immune parameters in the Nanostring expression analysis (Fig. 5c, d). In contrast, uninfected mice of both genotypes always cluster together (Fig. 5d; Additional file 1: Figure S7a, b, c).

**Fig. 6:**
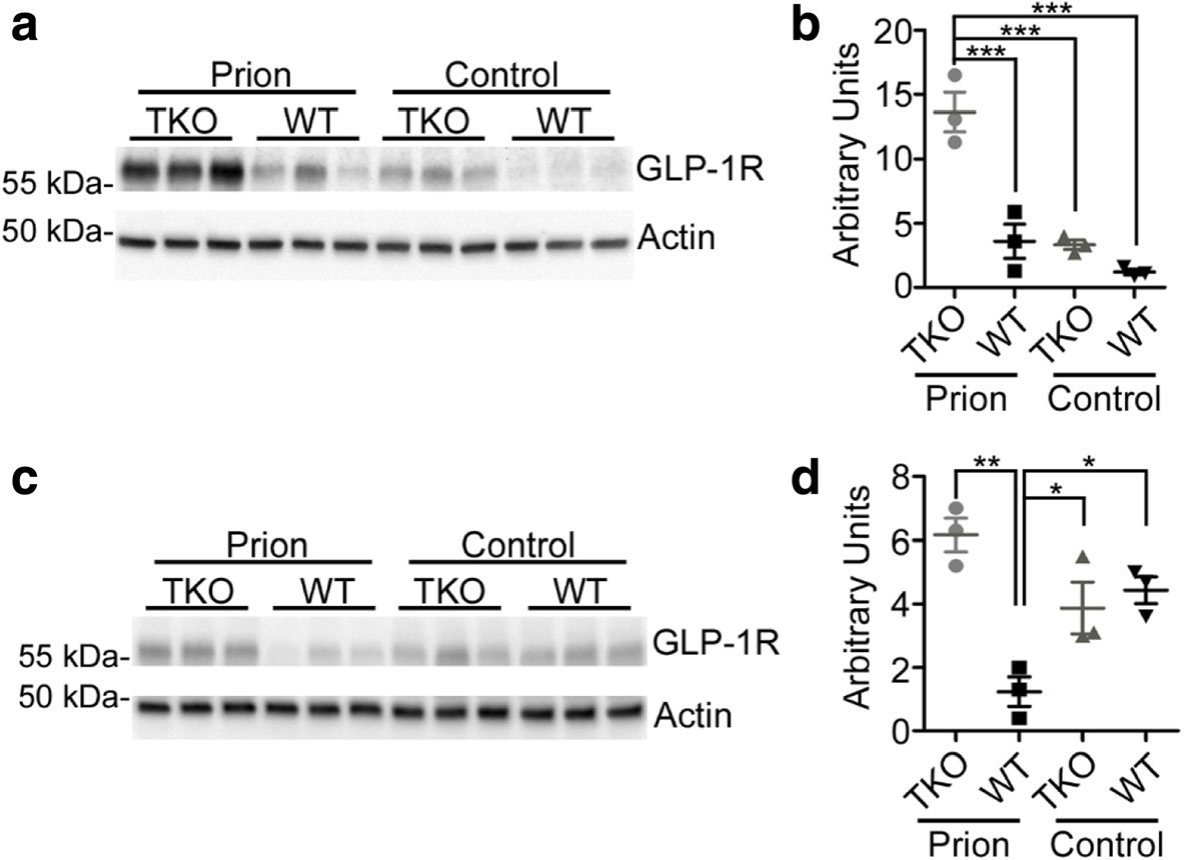
GLP-1R is upregulated in TKO-mice but not in WT-mice after prion infection **a** Western blot analysis of GLP-1R at clinical prion disease and age-matched controls (n = 3 individual mice per group). **b** Quantification of GLP-1R levels normalized to β-actin showed that GLP-1R is significantly increased in TKO-mice at clinical prion disease (p = 0.0001); n = 3 independent animals per group. **c** Western blot analysis of GLP-1R at day 80 p.i. and age-matched controls (n = 3 individual mice per group). **d** Quantification of GLP-1R levels normalized to β-actin showed that GLP-1R is increased in TKO-mice at day 80 post infection, but significantly downregulated in WT-mice (*p* = 0.0022); n = 3 independent animals per group

Recently, the involvement of A1-astrocytes in a mouse model of Parkinson’s disease has been described. Blocking of A1-astrocyte conversion by microglia was neuroprotective in this model [63]. Interestingly, up-regulation of the microglial glucagon-like peptide-1 receptor (GLP-1R) was identified as a disease marker in Parkinson’s disease and targeting of GLP-1R with an agonist lead to its down-regulation and the abolishment of microglia-dependent A1 astrocyte conversion [63]. To determine, if GLP-1R up-regulation also plays a role in prion diseases, and is targeted by our knockout strategy, we assessed levels of GLP-1R in brain homogenates from terminally prion diseased TKO- and WT-mice and their non-infected age-matched controls. Expression levels of GLP-1R were similar and low in uninfected mice (Fig. 6a, b). Surprisingly, GLP-1R was not upregulated in terminally prion diseased WT-mice compared to control mice (Fig. 6a, b). Since GLP-1R is considered to be a microglia marker [63], we propose that microglia dysregulation in prion diseases might be very distinct from that in other neurodegenerative diseases. Interestingly, GLP-1R was significantly increased in infected TKO-mice, pointing to a distinct microglia activation state as compared to infected WT-mice (Fig. 6a, b).

### Microglia homeostatic signature is lost early in the disease course in triple-KO mice upon prion infection

**Fig. 7:**
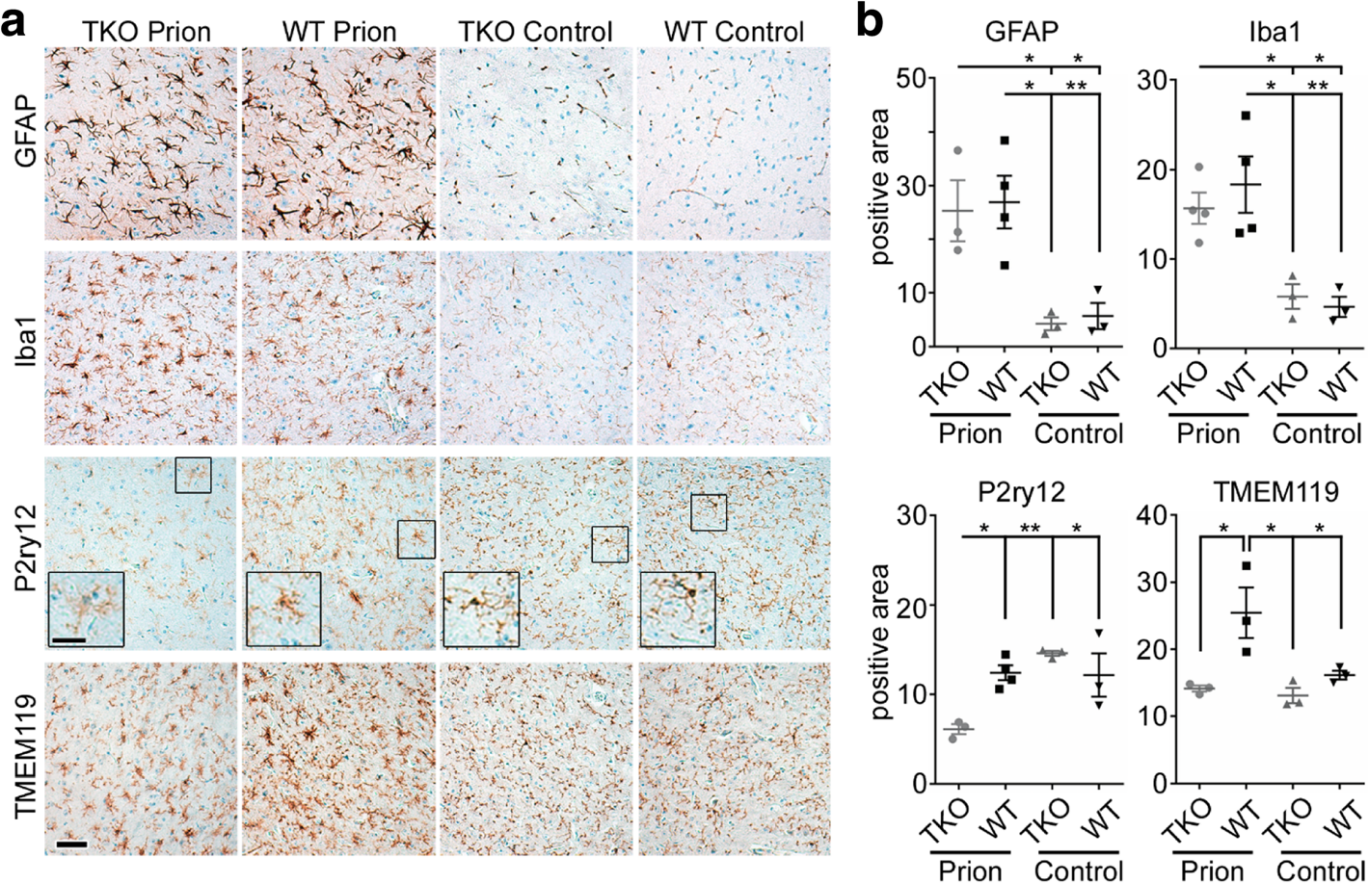
Homeostatic microglia markers are significantly altered in pre-clinical prion diseased TKO-mice at day 80 p.i. a Representative immunostaining of GFAP, Iba1, P2ry12, or TMEM119 in thalamus in brain sections of TKO- and WT-mice and age matched control; scale bar: 50 μm; high magnification: 25 μm. b Quantification of positive staining area (μm^2^ × 1000) of GFAP, Iba1, P2ry12, and TMEM119 of brain sections at 80 days post infection. While GFAP and Iba1 staining intensities are unchanged between prion infected TKO and WT-mice, both are significantly upregulated compared to uninfected control (GFAP *p* = 0.0058; Iba1 *p* = 0.0030). In contrast, P2ry12^+^ and TMEM119^+^-microglia are significantly dysregulated in prion infected TKO-mice only (P2ry12 *p* = 0.0069; TMEM119 *p* = 0.0089) n = 3–4 individual mice/group

Since GLP-1R was found to be significantly changed in TKO-mice at clinical prion disease, we asked whether microglia homeostasis was disturbed earlier in the prion infected TKO-mice. When we determined amounts of GLP-1R at day 80 post infection, we found it already upregulated in TKO-mice, but significantly down-regulated in prion infected WT-mice (Fig. 6c, d). Disease associated microglia activation is very high in prion disorders and starts rather early in disease in prion mouse models [22, 34]. Moreover, loss of homeostatic signature is especially severe in mouse models and human prion diseases [33, 36, 49]. Therefore, we took a closer look at the disease profile at day 80 and 110 post infection. We first determined the spongiosis score in infected mice. Although there was a trend towards more spongiosis in the infected TKO-mice at day 80, this was not significant (*p* = 0.1901) (Additional file 1: Figure S8). To assess neuropathological changes in the experimental groups, we stained brain sections of all four groups of mice at 80 days p.i. against pan-astrogliosis (GFAP) and pan-microglia/monocyte markers (Iba1) as well as microglia homeostatic marker proteins TMEM119 and P2ry12 (Fig. 7a). High magnification images confirmed that microglia morphology changed towards an activated phenotype with bushy appearance in both infected WT and TKO-brains (Fig. 7a). However, P2ry12 and TMEM119 intensity was more down-regulated in infected TKO- than in WT-brains. Quantification of total positive signals or cell counts revealed that while the pan-markers were unchanged at day 80 post infection, microglial homeostasis markers were significantly downregulated in prion infected TKO-mice in contrast to the WT-animals (Fig. 7b, Additional file 1: Figure S9).

Although A1-astrocytes are considered to be rather detrimental to neuronal health, our findings show for the first time that C3^+^-PrP^Sc^-reactive-astrocytes, a prion-induced specific subtype of reactive astrocytes, might act beneficially in prion disease pathophysiology by stimulating and supporting microglia and possibly slowing the progression of prion infection throughout the brain. Abolishment of this type of astrocytes does not seem to be a therapeutic option in prion disease treatment. It is unknown by which mechanism this occurs, but it will be an avenue of intense future investigation. The crosstalk between glia cell populations might be more subtle in prion diseases and seem to be very different from that in other neurodegenerative diseases.

## Discussion

Glia cells are getting increasingly recognized as active participants in the pathogenesis of neurodegenerative diseases [1, 26, 51]. However, only recently, it was proposed that microglia and astrocytes cooperate closely to generate a specific subset of astrocytes, designated A1 that is supposed to be more neurotoxic and might therefore considerably contribute to neuronal loss and disease progression [39]. Abolishment of A1-astrocyte formation by knockout or pharmacologic inhibition of TNF-α, IL-1α and C1qa have been shown to have therapeutic potential [39]. Since both, activation of microglia and massive astrogliosis are prominent in brains of human CJD patients, and are also reproduced in mouse models of prion infection, we set up the first study to investigate astrocyte profiles and their impact on prion disease pathophysiology. We show that complement 3^+^-PrP^Sc^-specific-astrocytes are highly abundant in human prion diseases and prion mouse models. A major aim of our study was to determine, if targeting these activated astrocytes could be used as a therapeutic strategy in prion disease treatment. Surprisingly, abolishment of C3^+^-astrocyte formation by knocking out TNF-α, IL-1α and C1qa accelerated the prion disease course.

Very recent investigations in mouse models of tauopathies and Alzheimer’s disease suggested regulation of the complement 3 receptor (C3aR), that is abundant on astrocytes and microglia, rather than C3 itself as a feasible target for therapies aiming to reduce gliosis and formation of A1-astrocytes. It could be shown that C3aR inhibition led to reversion of an immune network deregulation including microglia and astrocytes [40]. Therefore, it would be interesting to determine, if C3aR is altered in prion diseases and could pose a more suitable target than the cytokine triad TNF-α, IL-1α and C1qa. Dysregulation of immune functions have already been shown to be prominent in prion diseases [12, 22, 29, 61]. Since stimulating the inflammatory phenotype of microglia in prion diseases did not influence disease pathophysiology [28], restoring microglia homeostasis might be a more suitable strategy. While we detected no difference of Iba1^+^-microglia activation or disease markers such as *Clec7a* in our TKO-model, the loss of microglia homeostasis markers (P2ry12 and TMEM119) early in disease correlated with accelerated disease progression without affecting PrP^Sc^ loads.

The deposition of endogenous misfolded protein species is a hallmark of several neurodegenerative diseases [21]. Interestingly, when compared to WT-controls, the amounts of misfolded PrP^Sc^ were unchanged in prion infected TKO-mice despite reduction of C3^+^-astrocyte formation. This is in contrast with a recent report of Yun et al. [63] where decreased amounts of C3^+^-astrocytes in a mouse model for Parkinson’s disease directly correlated with decrease of α-synuclein deposition, reduction of neuronal loss, and improvement of disease outcome. In their study, the authors showed that increased microglial GLP-1R signaling was involved in the formation of A1-astrocytes and used the pharmacological targeting of GLP-1R to ameliorate microglia signaling and reactive astrocyte formation. Since Yun et al. identified upregulated GLP-1R on activated microglia in disease [63], we were interested to evaluate its therapeutic potential in prion diseases. Unexpectedly, GLP-1R expression was even reduced in prion infected WT-mice compared to control. Therefore, GLP-1R reducing strategies might probably be unsuitable as a therapeutic option in prion diseases. Interestingly, GLP-1R was significantly increased in the TKO-mice upon prion infection, stressing the fact that microglia are significantly dysregulated in the TKO-mouse model upon prion infection.

Although we speculated that early dysregulation of microglia in prion infected TKO-mice might be due to disturbed glia communication and reduction of C3^+^-astrocytes, we cannot rule out that dysregulated microglia phenotype is induced by the TNF-α, IL-1α and C1qa knockout itself. TNF-α, IL-1α and C1qa knockout mice develop normally and do not show signs of neurodegeneration [39]. Of note, TNF-α, IL-1α and C1qa are upregulated in mouse models of prion disease [12, 29] and TNF-α and IL-1α in human CJD [42], which would make polarization of astrocytes towards A1 very likely in prion diseases [39]. Knockout or depletion of either TNF-α or C1qa in mouse models of prion disease did not influence prion disease course after intracerebral prion infection [32, 43], however, microglia homeostatic phenotypes had not been determined in these studies. Interestingly, in both models, knockout significantly improved disease outcome after peripheral prion infection. In contrast, the impact of knockout of IL-1α or all three cytokines in prion disease pathophysiology has never been studied before. However, since we did not detect changes in PrP^Sc^ amount, which we assume, would be altered if microglia’s function phagocytosis would be impaired, direct effects of triple cytokine knockout on microglia might be mild. Regardless, if the effect in our TKO model was due to altered microglia response or reducing amounts of C3^+^-astrocytes, unfortunately, it did not ameliorate disease.

Microglia are the professional phagocytes of the brain and have been proposed to contribute to phagocytosis/degradation of PrP^Sc^ [3, 65]. The role of astrocytes in prion disease pathophysiology is less clear: Astrocytes have also been shown to efficiently degrade misfolded PrP species in vitro [13]. On the other hand, astrocytes express measurable amounts of the substrate protein PrP^C^ for conversion into PrP^Sc^ [48] and they have been shown to accumulate PrP^Sc^ [17, 62]. Astrocytes have also been suggested to actively replicated PrP^Sc^ and contribute to PrP^Sc^ production and disease progression [16, 37] or even to spreading of PrP^Sc^ [27, 60]. Since most of these studies have been performed in vitro, it is still controversial, if astrocytic PrP^Sc^ formation alone is sufficient to mount a clinical prion disease in vivo [2, 30, 44, 54]. Astrocyte subtypes might contribute differentially to the features ascribed to astrocytes in prion diseases with C3^+^-PrP^Sc^-reactive astrocytes potentially acting beneficial as suggested by our study. When we investigated the astrocyte expression profile in more detail, we could not determine a clear A1 profile. This might be attributed to specific activation patterns unique to astrocytes in prion diseases. However, our analyses come with several limitations: (I) We used bulk tissue from thalamus. Therefore, regional differences in astrocyte profiles in response to PrP^Sc^ deposition will not be considered since the thalamus is already very heterogeneous in terms of prion pathology. In contrast, Shi et al. could determine a slight clustering of A1-specific expression changes using microfluidic qPCR in bulk tissue analyses from brains of Tau transgenic mice [58]. (II) Most genes are not completely astrocyte specific and the astrocyte profile might be masked by down/upregulation of expression in other cells types. (III) We analyzed tissue at terminal disease, where a lot of cells are already exhausted, including microglia with a loss of functional signature [49]. (IIII) Only three animals were analyzed per group, which gives great variances with one outliner, which we had in both groups of infected animals. Nevertheless, we could see clear clustering into separate expression signatures. As it has been shown for myeloid cell populations in the neuroinflammatory brain [31], future research using single cells sequencing of astrocyte populations in preclinical and clinical animals will undoubtedly help to identify full picture of astrocyte profiles in the prion diseased brain. A better understanding of astrocyte subpopulations might help to determine regional activation pattern in the future [4, 47]. Moreover, stem-cell derived co-cultures have been shown to be feasible models to study aspects of neurodegeneration in the past [25]. The newly established in vitro model of prion infection and PrP^Sc^ replication in human astrocytes derived from induced pluripotent stem cells (iPS cells) might facilitate to study the capability of subtypes of astrocytes in prion disease pathophysiology in more detail [37]. Using advanced co-culture models might facilitate to dissect contributions of different cells types in future experiments [53].

Both, microglia and astrocytes are massively dysregulated in human and mouse prion disorders, but it remains poorly understood, how both cell types interact and contribute to progression of disease. Although C3^+^-PrP^Sc^-specific-astrocytes are abundant in human and mouse prion diseases, abolishment of their formation led to an acceleration of prion disease progression. Our data showed that astrocyte signatures in prion diseases are distinct from other neurodegenerative diseases. These include those found in response to chronic neurodegenerative diseases like Alzheimer’s and Parkinson’s diseases or aging [8, 15, 58, 63], and those stimulated following acute injuries and traumas [5, 14]. However, astrocyte responses are highly heterogeneous and not a simple ‘positive’ or ‘negative’ response to such complex mediators. Although A1-astrocytes have been shown to have largely detrimental effects in models for chronic diseases like Alzheimer’s, it has been hypothesized that this function could locally have a positive effect (e.g. removal of aberrantly firing neurons), while globally being detrimental (e.g. death of too many neurons) [38, 39]. Irrespective of the reason for the formation of this reactive subtype, future investigations into how astrocytes and microglia communicate in the face of such challenges will hold much hope for understanding a wide array of CNS diseases.

## Conclusion

Our study demonstrates that the expression signature of reactive astrocytes in prion diseases is very distinct from other neurodegenerative diseases and characterized by upregulation of complement 3 and a mixed A1/A2 phenotype. Unexpectedly, abolishment of C3^+^-astrocyte formation by specific cytokine knockout led to an acceleration of disease and early dysregulation of microglia homeostatic marker expression. Our findings rather exclude the abolishment of reactive astrocytes as a therapeutic option in prion disease treatment, while restoring microglia function might be a better option.

## Additional file

Additional file 1: 'Complement 3+-astrocytes are highly abundant in prion diseases, but their abolishment led to an accelerated disease course and early dysregulation of microglia' Supplementary Figures and Table. (DOCX 15895 kb)
