# Supplementary material for: Complement 3+-astrocytes are highly abundant in prion diseases, but their abolishment led to an accelerated disease course and early dysregulation of microglia
Source: Acta Neuropathol Commun. 2019 May 22;7:83. doi: 10.1186/s40478-019-0735-1 (PMC6530067; doi:10.1186/s40478-019-0735-1)
Supplement: Supplementary file 1 — 'Complement 3+-astrocytes are highly abundant in prion diseases, but their abolishment led to an accelerated disease course and early dysregulation of microglia' Supplementary Figures and Table. (DOCX 15895 kb) [file 40478_2019_735_MOESM1_ESM.docx]

**Supplementary Figures:**

**
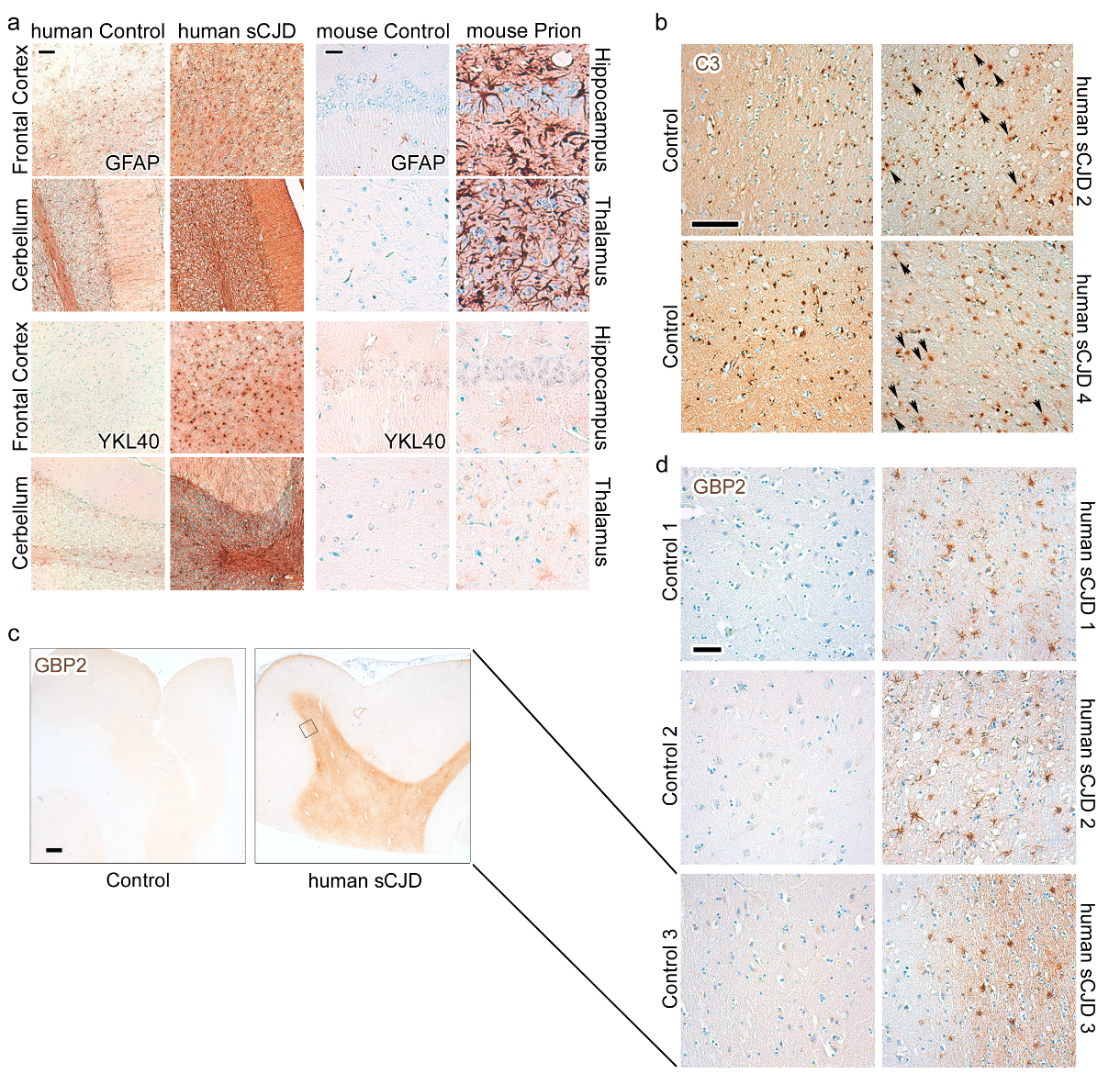
**

**Supplementary Figure 1** A1 astrocytes are highly abundant in human prion diseases **a** Pan-astrocytic markers GFAP and YKL-40 are highly enriched in mouse and human prion diseases; scale bar human: 100µm, mouse: 20µm. **b** Representative immune-histochemical staining with antibody against complement 3 in human sCJD cases. Although back ground staining is visible, C3-positive astrocytes (black arrows) could only be detected in sCJD-cases and not in controls; scale bar: 100µm. **c** Representative frontal cortex sections of control or sCJD individuals show that GBP2-positive A1 astrocytes can be only detected in human prion disease but not in age matched control brains; scale bar: 1mm. **d** Close up of GPB2‑positive area in sCJD as shown in figure **1c** in frontal cortex sections of individual sCJD cases and healthy controls confirmed A1-astrocyte specific GBP2 immunoreactivity only in sCJD brains; scale bar: 50µm.

**
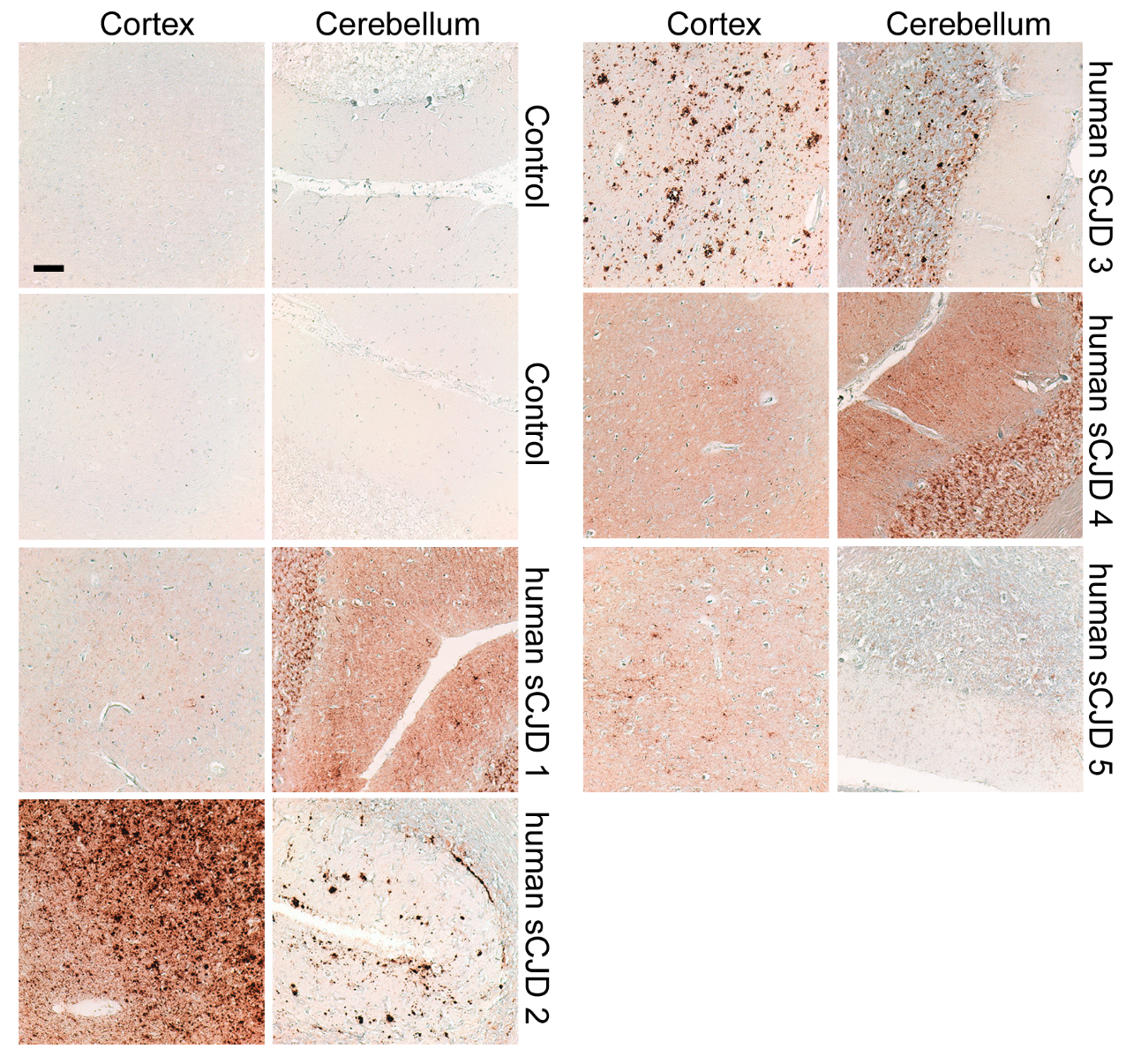
**

**Supplementary Fig. 2** Representative immunohistochemical staining of PrP^Sc^ deposition pattern in frontal cortex and cerebellum of the sCJD cases investigated in this study (n=5 individual cases) and two of the age matched controls. Note that amount of PrP^Sc^ deposition does not correlate with abundance of A1 astrocytes; scale bar: 100µm.


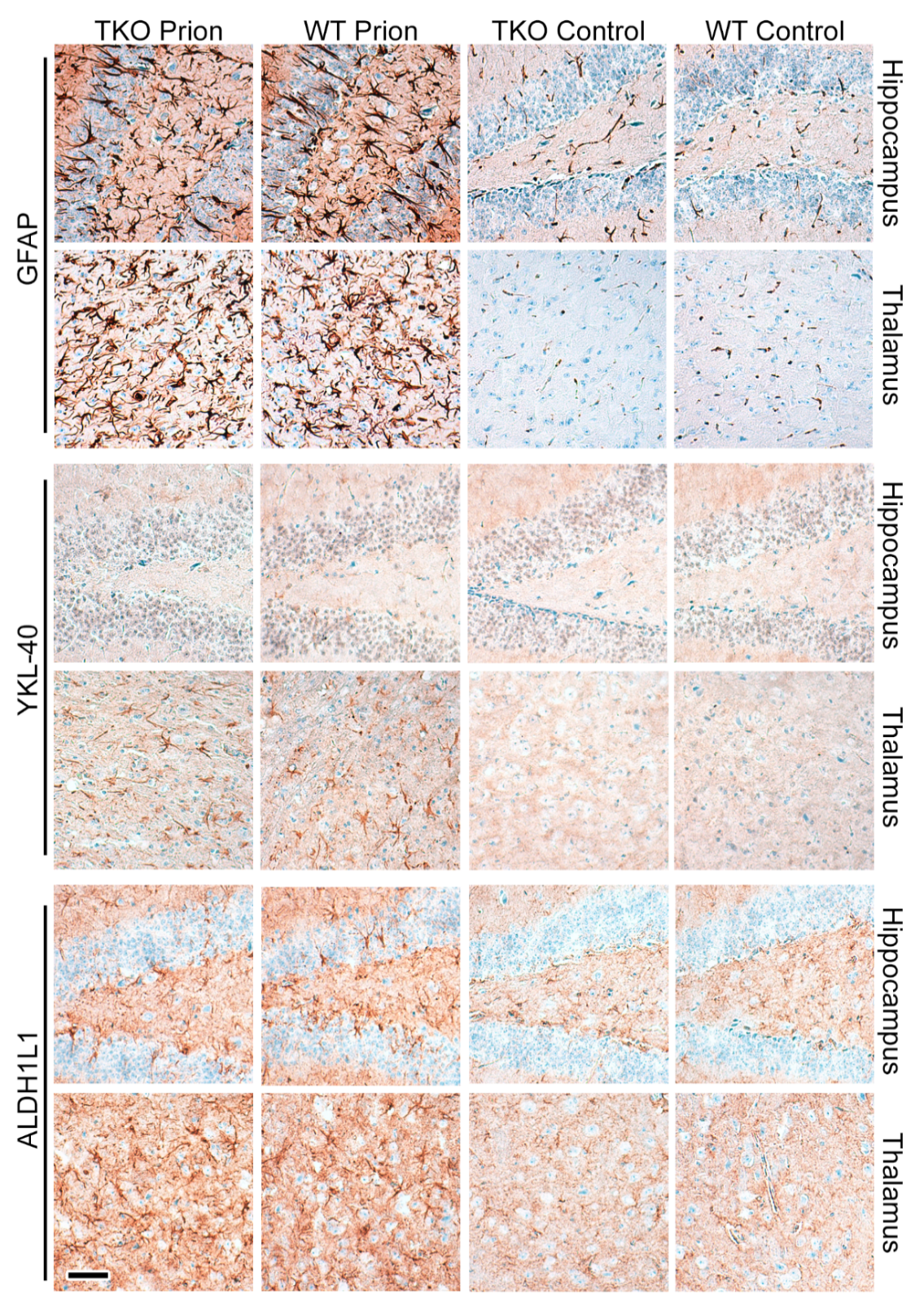


**Supplementary Fig. 3** Astrocyte staining in terminal prion disease: Staining intensity of astrocyte markers GFAP, YKL40 and ALDH1L1 is increased upon clinical prion disease, but similar in clinical TKO mice (TNF‑α, IL‑1α, and C1qa Triple-KO mice) in comparison to clinical WT animals. Representative immuno-staining of GFAP, YKL and ALDH1L1 in hippocampus and thalamus of brains of clinical prion diseased mice and age matched uninfected controls; n=3 individual mice per group; scale bar: 50µm


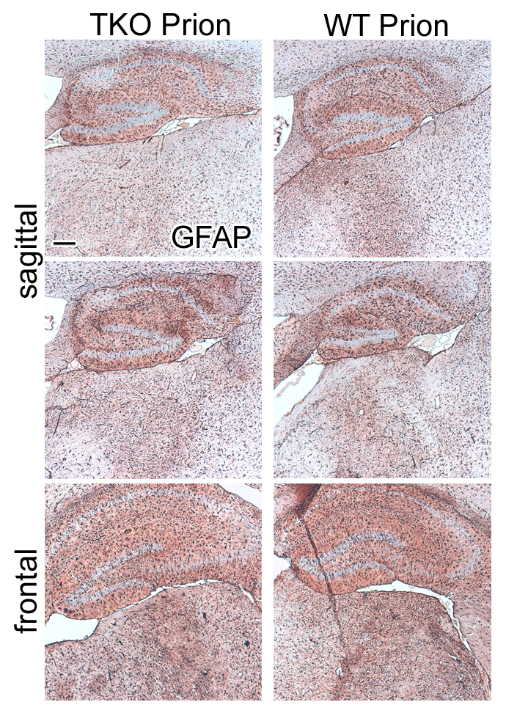


**Supplementary Fig. 4** Pan-astrocyte activation marker GFAP is unchanged in clinical TKO mice (TNF‑α, IL‑1α, and C1qa Triple-KO mice) when compared to clinical WT-mice. Overview spanning parts of the cortex, hippocampus and thalamus of representative staining of individual prion infected terminally sick WT or TKO-mice showed no differences in GFAP immuno-reactivity; scale bar: 200µm.


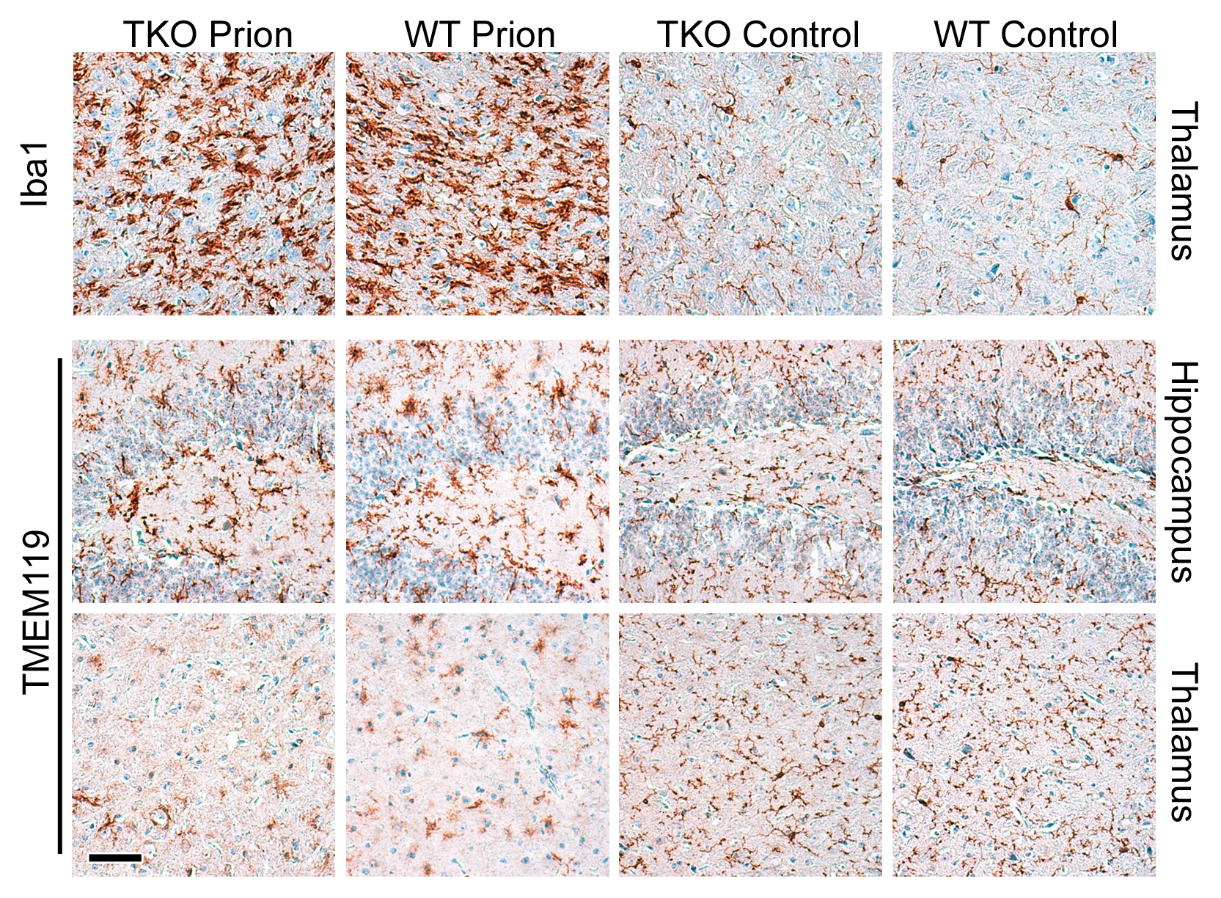


**Supplementary Fig. 5** Microglia staining in terminal prion disease: Abundance of microglia marker Iba1 is increase upon clinical prion disease but similar in clinical TKO mice (TNF‑α, IL‑1α, and C1qa Triple-KO mice) in comparison to clinical WT animals. Staining of microglia homeostasis marker TMEM119 show activated dysregulated phenotype of microglia in terminal prion disease with partial loss of immune-reactivity especially in Thalamus. Again, there is no difference in prion infected TKO- versus WT-mice. Representative immuno-staining of GFAP, Iba1 and TMEM119 in hippocampus and thalamus of brains of clinical prion diseased mice and age matched uninfected controls; n=3 individual mice per group; scale bar: 50µm.


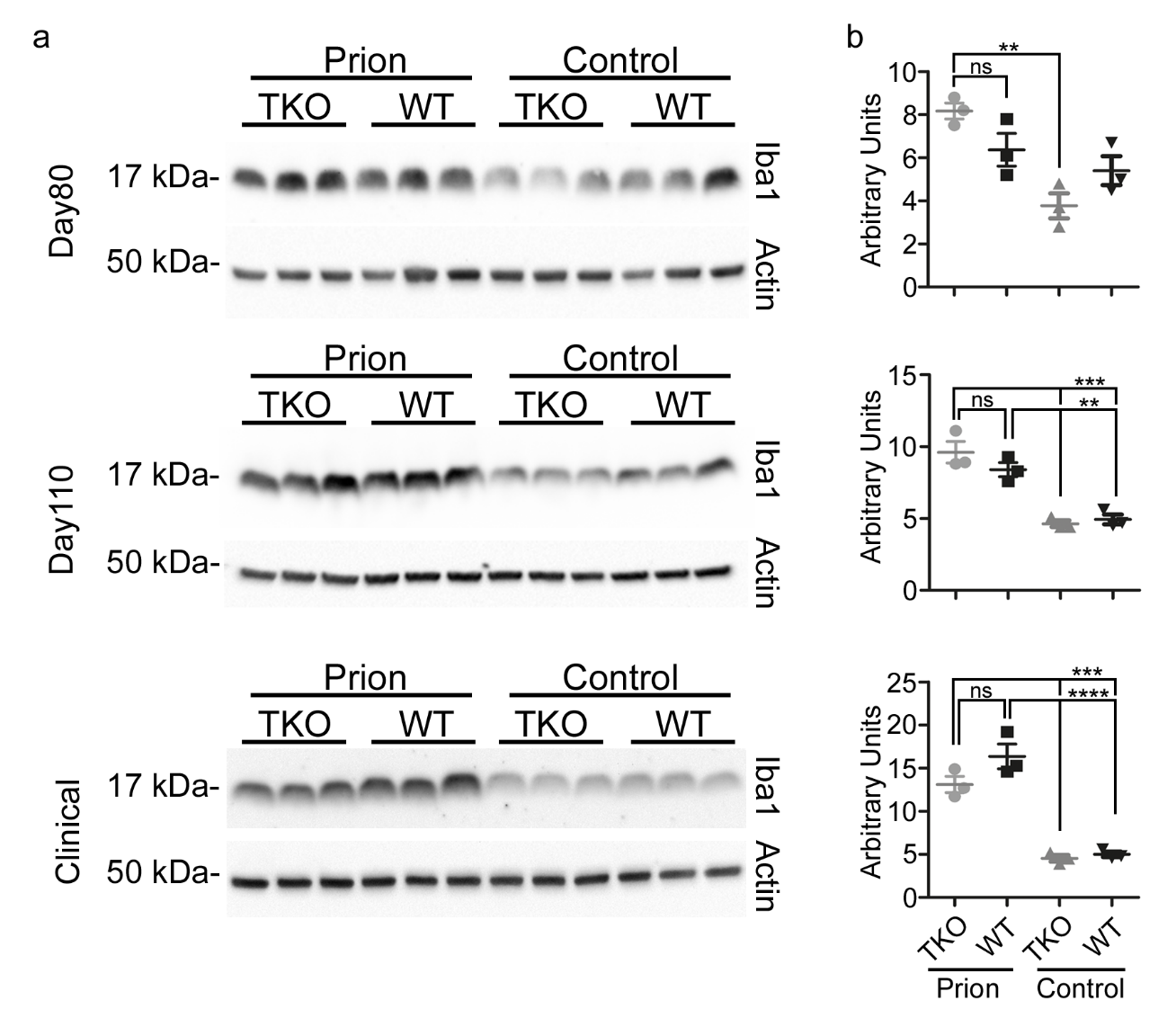


**Supplementary Fig. 6** Western blot analysis show abundance of the microglial/monocyte marker Iba1 at day 80 and day 110 p.i. and clinical prion disease (n=3 individual animals per group) **a** and where quantified **b**  and normalized to ß-actin expression. Shown is the mean expression +/- SD. Although Iba1 abundance is significantly increased in prion diseases at late and clinical time points, we could not detect differences between TKO‑ (TNF‑α, IL‑1α, and C1qa Triple-KO mice) and WT‑mice.


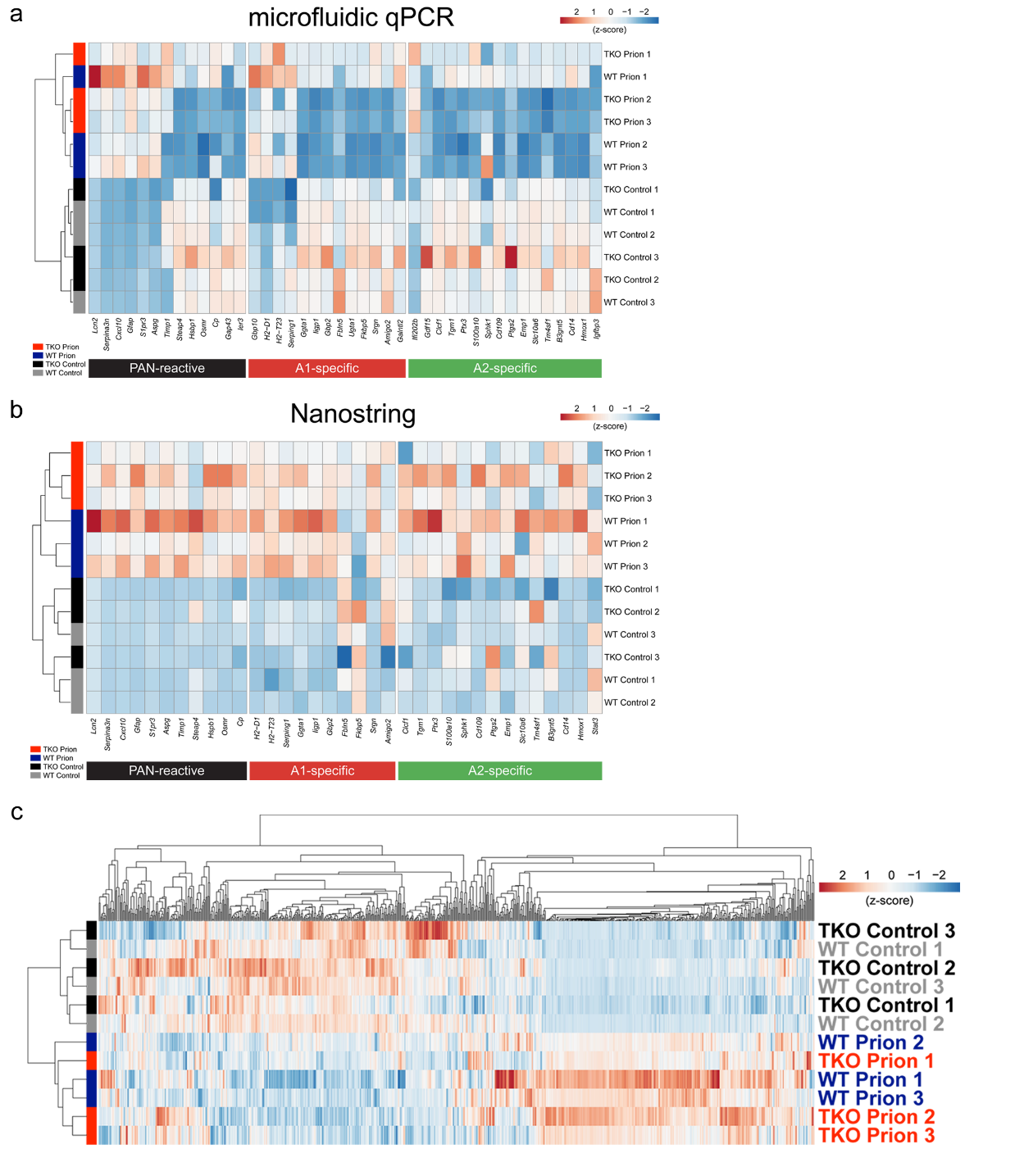


**Supplementary Fig. 7** Expression signature of astrocytes in prion diseases is characterized by a specific mixed activation type with PAN reactive, A1-specific, and A2-specific gene activation. **a** Microfluidic qPCR for activated astrocytic genes in terminally sick mice and age matched controls grouped into. **b** Nanostring nCounter analysis for activated astrocytic genes in terminally sick mice and age matched controls grouped into PAN reactive, A1-specific, and A2-specific genes. **c** Heatmap of all transcripts from Nanostring encounter expression analysis. Prion infected mice distinctly separate from non-infected animals. N=3 individual mice per group for all analyses.


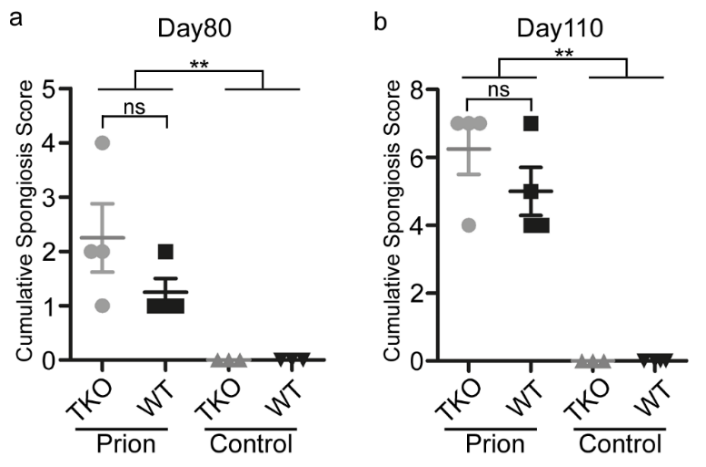


**Supplementary Fig. 8** Degree of spongiosis is similar at preclinical time points. Semi-quantitative assessment of the spongiosis score in cortex, hippocampus, thalamus, and cerebellum at **a** preclinical day 80 or **b** day 110 was plotted as cumulative value. Although there was a trend towards more spongiosis in the TKO‑mice (TNF‑α, IL‑1α, and C1qa Triple-KO mice), it was not significant (day 80 p=0.1901; day 110 p=0.2708).


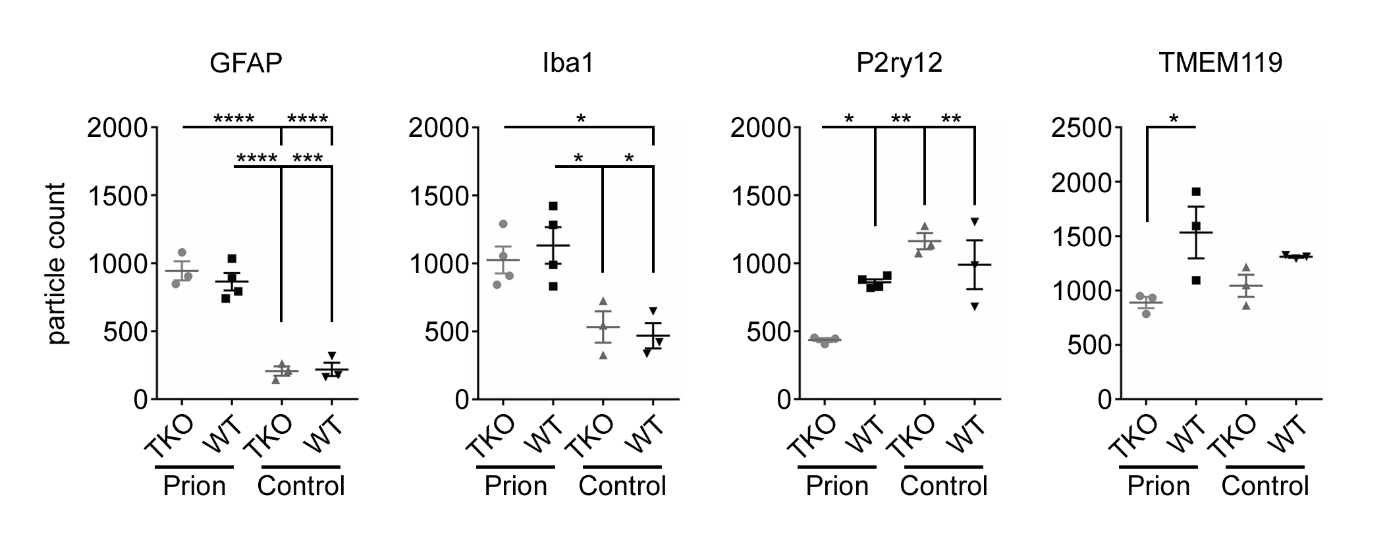


**Supplementary Fig. 9** Quantification of positive particle count of GFAP, Iba1, P2ry12, and TMEM119 staining on brain sections at 80 days post infection (Figure 6). While GFAP and Iba1 counts are unchanged between prion infected TKO- (TNF‑α, IL‑1α, and C1qa Triple-KO mice) and WT-mice, both are significantly upregulated compared to uninfected control (GFAP p<0.0001; Iba1 p=0.0044). In contrast, particle counts of P2ry12^+^ and TMEM119^+^-stained microglia are significantly dysregulated in prion infected TKO-mice only (P2ry12 p=0.0019; TMEM119 p=0.0362). Particles were included in the quantification only when their area was larger than 10 μm^2^.

|  | **Individual incubation times** | **Mean incubation time** |
| --- | --- | --- |
| **WT-mice** | 142, 143, 144, 146, 147, 147, 148, 159 | 145.9 +/- 2.7 |
| **TKO-mice** | 121, 127, 128, 129, 131, 131, 133, 135, 136, 137 | 130.8 +/- 4.8 |

**Supplementary Table 1:** Individual incubation times to terminal disease
